# Gynecology

**Published:** 1893-12

**Authors:** 

**Affiliations:** F. R. C. S., Ed., Professor of Anatomy University of Texas; late Physician for Diseases of Women, Edinburgh Providence Dispensary


### DEPARTMENT OF GYNECOLOGY.

EDITED BY WM. KEILLER, F. R. C. S., ED.,
Professor of Anatomy University of Texas; late Physician for Diseases of Women, Edinburgh Providence Dispensary.

Papillary Cyst of the Paro-ophoron.—Bland Sutton records the following case: Female patient, aged 41, single; had ceased to menstruate for five years. Abdomen contained a fluid not due to heart, kidney or liver affection. A soft mass could be felt in the pelvis by rectal examination. Exploratory incision revealed the left ovary replaced by a large soft warty mass the size of two closed fists; right ovary similar but smaller. Both were removed and abdomen flushed. Pelvic peritoneum covered by soft pink velvety warts which bled freely when sponged. Abdomen closed. Uninterrupted recovery.

From this and other cases he concludes:

1. The tumor was a paro-ophoritic cyst which had fungated through its sac walls. Both ovaries are frequently affected.

2. These cysts are usually associated with hydroperitoneum, and the fluid is very rich in cells.

3. They sow warty growths broadcast over the peritoneum, which proliferate mostly in the pelvis, the cells gravitating there.

4. They are not malignant, and if the parent tumor or tumors be removed, the general warty condition of the peritoneum will quickly disappear (compare the appearance and disappearance of warts on the hands).

5. The cells in the fluid of any case of hydroperitoneum due to a tumor cannot be considered as indicating malignancy.

He calls attention to a rare case of papilloma of the pelvis of the kidney, accompanied by secondary villosities in the bladder, and insists that in all post mortem of villous bladder the kidneys should be examined for papillomata.--*Lancet*, (*London*), April 8, 1893.

---

FIFTY-NINE CONSECUTIVE OPERATIONS FOR SUPPURATING PERIUTERINE LESIONS.—Terrier & Hartman, Paris, *Review de Chirurgie*, May, 1893:

Suppuration bilateral in 24 cases.

Right side only, 16 cases.

Left side only, 16 cases.

Intraperitoneal, but of undetermined side, 1 case.

Of the 32 unilateral cases the appendages of the opposite side were absolutely healthy in six cases; diseased in twenty-six cases.

*Origin of Suppuration.*—In one case there was an encysted intraperitoneal suppuration opening into the rectum; in forty-two cases it originated in connection with the tube; in three cases the abscesses were purely ovarian, in one case there were multiple foci. Most cases occupied the ampulla of the tube (17), seven were tubo-ovarian, ten tubo-intestinal.

*Mortality after Operation.*—In all the cases operated on, 11.86 per cent. Since the operators have operated with elevated pelvis (Trendelenberg's position) their mortality has been reduced from 17.8% to 5-17%, due to the possibility of operating by sight in this position, whereas one has to operate blindly in the ordinary supine posture. This last mortality of 5.17% is much superior to Segoud's mortality of 9.2% in his 102 cases of hysterectomy for similar lesions.

*Remote Results.*—Forty-six cases followed up. One case operated on in 1887 has had no more trouble. Of three operations in 1888, nine in 1889, nine in 1890, seven in 1891 and sixteen in 1892, forty-two have had no more trouble, two suffer considerable, two have a little pain occasionally; there is no one that is not improved. As to menstruation, twenty-two have never menstruated, six are regular, three are a little irregular, three have a period every three or four months, two have had metrorrhagia, necessitating secondary curetting, one has rather more profuse periods than normal.

The surgeons advocate laparotomy rather than vaginal hysterectomy for these cases. In all their cases they have had no hernia since using buried sutures for each successive layer of the abdominal wall, except in one patient, who developed a bronchopneumonia immediately after the operation.

They have never been unable to operate from the abdomen, and have never required a secondary hysterectomy.

In one or two cases they have had persistent stercoral fistules, but these occur also in vaginal operation.

In tubo-ovarian suppuration laparotomy gives good permanent results, and being a less grave operation and permitting in suitable cases the preservation of healthy appendages where one side is free from disease, it is to be preferred to hysterectomy.

Certain cases of pelvic suppuration, however, may be treated by simple vaginal incision, and in very diffuse suppuration vaginal hysterectomy may be practiced.

---

Tardy Labor Due to Softness of the Fœtal Cranium.—In the *Loire Medicale* Dr. Blanc calls attention to a parchment-like condition of the flat bones of the fœtal cranium, owing to which the head acts inefficiently as a wedge at the pelvic floor, as being a hitherto undescribed cause of delay in labor. The bones give a parchment-like crepitation to the examining finger. Forceps are indicated.

The editor of this department feels special interest in this, having had a case precisely answering the description. Forceps ended successfully a tardy labor.

---

PELVIC ABSCESS.—C. I. Cullingworth, *Lancet* (London), Nov. 4, 1893. In the inaugural address delivered before the Midland Medical Society, Birmingham, Dr. Cullingworth says, that as in other parts of the body, we now know that suppuration in the pelvis cannot be idiopathic, but must always be due to infection. With modern knowledge of pelvic pathology, a more definite diagnosis than simply "pelvic abscess" should usually be possible.

1. *Connective Tissue Abscess.*—He quotes extensively from Prof. Keiller's paper on pelvic cellulitis and peritonitis (*Amer. Jour. Obstet. and Dis. Women*, Sept., 1893, and Trans. Texas State Med. Association, 1983). Agreeing with him in likening inflammation of the pelvic cellular tissue to that of the back of the hand following a septic wound of the palm. These exudations are usually limited by the peritoneal attachments to one side of the pelvis. The usual cause is laceration of cervix or vagina during labor, the latter usually due to forceps delivery. Also operation on the cervix or vagina, in each case, of course, sepsis being added. Abortion rarely causes cellulitis where there is no instrumental interference.

He also agrees with Dr. Keiller's paper as to the importance of the role played by the pelvis lymphatic vessels and glands in the production of pelvic abscess.

Such abscesses point oftenest on the anterior abdominal wall, forming a strip of induration just above and parallel with Poupart's ligament. They may rarely point above the iliac crest, above the pubic symphisis, or at the umbilicus; or, they may follow the femoral artery beneath Poupart's ligament, or the gluteal or sciatic vessels to the gluteal region. Dr. Cullingworth has never had a cellulitic abscess open into vagina, rectum or bladder. He believes that most abscesses opening into mucous tracts are tubal or ovarian (or peritonitic, secondary to tubal or ovarian affections).

The treatment of pelvic cellulitic abscess is incision and drainage. In these cases abdominal section is uncalled for. Once opened they tend to rapid recovery. They do not interfere with subsequent pregnancy. He believed that most fluctuating swellings depressing the vaginal vault are not cellulitic and should not be attacked from the vagina.

Of *thirty-three cases of pelvic abscess, not cellulitic*, thirty-seven were due to purulent salpingitis; thirteen to purulent salpingitis with suppurating cyst of the ovary; eleven to suppurating cyst of the ovary; seven to tubercular disease of ovary, tube, or both; one to disease of the vermiform appendix; one to suppurating lumbar gland, and six in which the cause was undetermined. Thus *purulent salpingitis* was the cause in 60% of the cases. Encysted collections of pus in Douglas's pouch are almost invariably merely complications of purulent salpingitis.

*Suppurating cysts of the ovary* is the next most common cause of suppurating pelvic peritonitis. Small suppurating ovarian cysts are not readily recognized by examination, and even on abdominal section are so obscured by adhesions as to escape detection as the source of the peritonitic abscess. Such cases are often diagnosed as pelvic cellulitis. The proper treatment is abdominal section and enucleation. Cullingworth believes most cases of suppurating ovarian cysts are secondary to salpingitis. Of thirty cases purulent salpingitis was found in thirteen, chronic salpingitis in twelve, ulceration in vermiform appendix in one, cause undetermined in four.

*In tubal or ovarian tuberculosis*, whether or not complicated by tubercular peritonitis, the best results may be expected from operation if the lungs be healthy.

*Pelvic fistulæ.*—Dr. Cullingworth quotes four cases of fistulæ dischaging pus into the rectum, vagina, cervix uteri, and bladder respectively, as representing typical cases. Two were due to unsuspected dermoid cysts which suppurated shortly after pregnancy,—the others due to tubal and ovarian disease. He urges that these cases of fistulæ communicating with the pelvic mucous tracts are not cellulitic, and that even the most complicated cases are successfully treated by abdominal section.

---

HYSTERECTOMY VERSUS HIGH AMPUTATION OF THE CERVIX FOR CANCER, T. Bowreman Jesset. (*Brit. Gynæc. Jour.*, May 1893.) Mr. Bowreman Jesset, the president of the British Gynecological Society, in his presidential address compares exhaustively the statistics of vaginal hysterectomy and high amputation of the cervix for cervical cancer.

The dangers of vaginal hysterectomy are: 1, Intestinal obstruction; 2, ligature or division of the ureters; 3, vesico-vaginal fistula; 4, peritonitis; 5, primary or secondary hemorrhage. None of these dangers threaten high amputation of the cervix.

The death rate in 1273 cases of hysterectomy in Europe and America averages 15%; in high amputation it is very low.

He concludes, that where the diseased area can be removed by supra-vaginal amputation of the cervix, it is much the preferable operation, and hysterectomy should be reserved for those cases where the body of the uterus is involved.

---

THE ETIOLOGY OF ECTOPIC GESTATION, I. C. Webster, *Edin. Med. Journal*, Nov., 1893. In a thoroughly scientific article on the causes of tubal pregnancy, Dr. I. C. Webster offers the following ingenious explanation of its occurrence, based on observations on the changes in tubes and uterus in normal and tubal gestation:

1. In the lowest animals possessing a genital tract there is no differentiation of tube and uterus, and pregnancy may occur in any part of the Müllerian duct.
2. As the result of impregnation, changes resulting in the formation of a decidua vera take place normally in the uterine cavity only.
3. Careful examination of the whole length of the tubes in normal pregnancy has revealed (but rarely) occasional limited areas of tube that have undergone a similar decidual change, the rest of the tube being unaltered.
4. An ovum may be fertilized in any part of the tubo-uterine canal.
5. Given an ovum fertilized in the upper part of a tube, if decidual changes take place below it, it may attach itself to that part of the tube, instead of passing on to the uterus.
6. Constricting bands, tubal inflammation, anything, in fact, retarding the passage of the ovum along the tube to the uterus, may act as predisposing causes.

He urges afresh the improbability of primary abdominal pregnancy and considers so-called ovarian as originally tubo-ovarian gestation.

